# Association of meteorological exposures with major cancer mortality in Minhang District, Shanghai: a time series study based on distributed lag non-linear models

**DOI:** 10.3389/fpubh.2026.1923874

**Published:** 2026-08-27

**Authors:** Jiaqi Guo, Junhui Huang, Chao Xu, Shuili Xuan, Jingyi Ni, Wei Liu, Linli Chen, Yibin Zhou

**Affiliations:** 1 Information Management Section, Shanghai Minhang District Center for Disease Control and Prevention (Shanghai Minhang District Health Supervision Institute), Shanghai, China; 2 Department of Tuberculosis Prevention and Control, Shanghai Minhang District Center for Disease Control and Prevention (Shanghai Minhang District Health Supervision Institute), Shanghai, China; 3 Department of Anesthesiology, Zhongshan Hospital, Fudan University, Shanghai, China; 4 Director's Office, Shanghai Minhang District Center for Disease Control and Prevention (Shanghai Minhang District Health Supervision Institute), Shanghai, China

**Keywords:** distributed lag non-linear model, environmental epidemiology, extreme weather, lag effects, lung cancer, major cancer mortality, meteorological exposures

## Abstract

**Objective:**

This study examined the associations between meteorological exposures and major cancer mortality, with a focus on exposure-lag relationships.

**Methods:**

We collected daily cancer death records and weather data from Minhang District, Shanghai, 2014 to 2023. Joinpoint regression estimated annual percent changes in age-standardized cancer mortality rates. Spearman's rank correlation was employed to explore how meteorological factors related to cancer death. Finally, a distributed lag non-linear model (DLNM) evaluated exposure-lag effects of meteorological factors on daily cancer death risk.

**Results:**

This study included 26,684 cancer deaths. Spearman correlation analysis showed no significant linear associations between meteorological factors and daily mortality. However, the DLNM revealed significant non-linear and delayed effects, with low temperature and low humidity showing increased risks at specific lag days. Over a 0–21-day lag period, extreme weather affected lung cancer death risk more strongly than colorectal or gastric cancer risk. When daily mean temperature dropped to its 2.5th percentile, the estimated relative risk (RR) was 2.807, with a 95% confidence interval (95% CI) of 1.412–5.579. At the same percentile of daily minimum temperature, the cumulative RR for lung cancer death was 3.386 (95% CI: 1.697–6.755). When relative humidity was at its 2.5th percentile, the RR was 1.690 (95% CI: 1.269–2.250). Low temperature and low humidity showed increasing cumulative death risk with longer lag times.

**Conclusions:**

Lower temperatures and humidity may raise major cancer mortality, especially for lung cancer. Focusing on extreme weather and early warnings could thus improve health management for high-risk groups.

## 1 Introduction

Malignant tumors stand as a primary contributor to premature death worldwide (1). They also place considerable strain on social stability and economic advancement. Within the Asia-Pacific region, the burden imposed by these diseases is notably heavier than elsewhere (2). In 2022, the global cancer mortality rate was 123.5 per 100,000 population, while China's rate reached 182.3 per 100,000, substantially exceeding the global average (3). When viewed together, these figures point to an urgent reality: slowing the upward trend in cancer deaths and lessening the burden carried by patients have become central challenges for cancer prevention and control.

In 2025, the World Health Organization (WHO) issued a forceful declaration: “the climate crisis is a health crisis.” According to one calculation, high temperature exposure alone accounts for roughly 546,000 deaths annually (4). Meteorological factors have received growing research attention in recent years regarding their impact on public health. These factors help regulate a range of physiological processes in the body. When weather conditions are favorable, they support a state of comfort; when conditions turn abnormal, they may set off illnesses or even prove fatal (5). Available evidence hints at a possible connection between meteorological variables and death from malignant tumors. For example, atmospheric pressure correlates meaningfully with lung cancer mortality (6). Similarly, sharp temperature fluctuations have been associated with elevated death rates among individuals with lung cancer (7). Nevertheless, the lagged health effects of meteorological exposures remain insufficiently studied.

DLNM simultaneously captures the non-linear association between exposure and response, as well as the temporal distribution of lagged effects. This approach overcomes the constraints of traditional linear and single-lag models and offers a more robust statistical framework for estimating how meteorological factors influence major cancer mortality over both short-term and delayed time windows (8, 9). As a district at the geographical center of Shanghai, Minhang has a subtropical monsoon climate, with four distinct seasons, rain and heat in the same season, and ample precipitation (10). Evidence on the acute mortality associations of meteorological exposures in regional populations remains scarce. Accordingly, we applied a DLNM to assess how short-term weather variables (e.g., temperature and humidity) relate to major cancer mortality among registered residents of Minhang District, Shanghai. This study will help elucidate the acute and delayed effects of meteorological factors on cancer mortality in a regional population, while also providing empirical evidence to support the design of targeted public health interventions, including community-based early warning systems and enhanced protective measures for vulnerable groups.

## 2 Materials and methods

### 2.1 Data collection

From the Shanghai Death Registration Information System, we extracted records of major cancer deaths among registered residents of Minhang District. The study captured all such fatalities occurring between January 1, 2014 and December 31, 2023. Any entries lacking complete demographic details or a clear cause of death were discarded. For each decedent, we extracted the following: identification number, sex, death date, underlying cause, and confirmed cancer diagnosis. All underlying causes of death in this study were coded by the Minhang District Center for Disease Control and Prevention according to the International Statistical Classification of Diseases and Related Health Problems, 10th Revision (ICD-10). Based on the mortality ranking during the study period, the three leading causes of cancer death were selected as the analytical targets, defined as: lung cancer (ICD-10: C33–C34), gastric cancer (C16), and colorectal cancer (C18–C20). Other malignancies (ICD-10: C00–C15, C17, C21–C32, C35–C97, excluding the three codes listed above) were included only for descriptive purposes and were not incorporated into the DLNM analyses.

Daily weather data for Minhang District, including daily mean, maximum, and minimum temperatures, relative humidity, precipitation, atmospheric pressure, and wind speed, were obtained from the Shanghai Meteorological Bureau for the period from January 1, 2014, to December 31, 2023.

Daily concentrations of air pollutants—including PM2.5, NO_2_, O_3_, PM10, S0_2_ and CO—during the study period were extracted from the public real-time data platform (https://www.cnemc.cn/sssj/) operated by the China National Environmental Monitoring Center (CNEMC), using the records for Shanghai. The CNEMC operates under the Ministry of Ecology and Environment of China and is the official provider of national ambient air quality monitoring data, which are widely used in environmental and epidemiological research.

### 2.2 Exposure assessment and data linkage

To link the meteorological data to individual death records, we adopted an index date-based matching strategy, which consisted of temporal and spatial matching procedures:

(1) Temporal matching: The date of death of each individual served as the index date. On this basis, the daily regional mean values of temperature, humidity, and pressure on the index date were assigned to all deaths occurring on that day. For the subsequent DLNM, we constructed a cross-basis matrix extending from the index date to the maximum lag days (e.g., lag 0–lag 21 days) to capture the delayed effects of meteorological exposures on mortality risk.
(2) Spatial matching: Given the regional-scale design of this study, we applied a district-wide uniform exposure assignment approach. Specifically, the daily meteorological values recorded at the Minhang District national basic meteorological station were uniformly assigned to all individuals who died within the district on the corresponding date. This approach was adopted for two reasons: (i) individual-level residential addresses were not available due to data privacy restrictions; and (ii) Minhang District covers a relatively small administrative area (approximately 372 km^2^) with limited spatial heterogeneity in meteorological conditions, making a single representative station acceptable for capturing the overall district-level exposure. No spatial interpolation was applied, as the study was conducted at the district level with a single monitoring station.

### 2.3 Ethics statement

This study was reviewed and approved by the Ethics Committee of Shanghai Minhang District Center for Disease Control and Prevention (approval number: EC-P-2024-014). All data were obtained from the Minhang District Center for Disease Control and Prevention and were fully de-identified prior to provision, ensuring that no personal identifiable information was accessible to the research team. As this was a retrospective observational study using pre-existing, de-identified secondary data, with no direct intervention or potential risk to human subjects, and given that obtaining informed consent from deceased individuals was impracticable, the ethics committee granted a waiver of informed consent.

This study was conducted in accordance with the Declaration of Helsinki and the relevant regulations of China on ethical review of biomedical research involving human subjects.

### 2.4 Statistical analysis

#### 2.4.1 Data description and correlation analysis

We used Excel 2019 to compile the mortality and meteorological datasets from Minhang District, Shanghai covering the years 2014 through 2023. We summarized continuous variables using mean (SD) or median (IQR), and categorical variables using counts and percentages (n/%). We plotted the trends in age-standardized cancer mortality rates using R. Joinpoint regression gave us the annual percent change (APC) in age-standardized cancer mortality rates across the study period. An APC above zero pointed to a rising trend, while a value below zero indicated a fall. To examine how closely meteorological factors correlated with cancer deaths, we turned to Spearman's rank correlation, setting the significance threshold at 0.05.

#### 2.4.2 Model construction

DLNMs (8) based on generalized additive models (GAMs) were constructed using R version 4.4.1 with the “dlnm” (11) and “mgcv” packages to investigate the lagged effects of various meteorological factors on cancer mortality. The basic form of the model is specified as follows:


$$\begin{array}{l} \text{lg}[\text{E}(Y_{t})]=\text{intercept}+\text{cb}(\text{factor},\text{ lag})\\ \;+\text{ns}(doy,7)+\text{ns}(time\_index,3)+\sin(2\pi \;doy/365)\\ \;+\cos(2\pi \;doy/365)+\text{ns}(\text{RH},\text{ df})+\text{ns}(\text{DP},\text{ df})+\text{Dow}\\ \;+\text{offset}(\log(pop_{t})) \end{array}$$

We defined time as *t*, with *Yt* and E(*Yt*) representing the observed and expected daily cancer death counts. A cross-basis function cb(.) was applied to characterize the three-dimensional exposure–lag–response relationship for each meteorological factor. The meteorological factors included daily mean temperature (*t*_avg), daily maximum temperature (*t*_max), daily minimum temperature (*t*_min), diurnal temperature range (*t*_gap), daily mean relative humidity (RH), daily precipitation (DP), daily mean atmospheric pressure (MP), and daily mean wind speed (WS). The maximum lag length was denoted as lag; The natural cubic spline function is denoted as ns(.); Day-of-year (doy, 1–365/366) was adjusted for using a spline with 7 degrees of freedom (df) to control within-year seasonal patterns (12); *time_index* is a sequentially increasing integer variable starting from day 1 (January 1, 2014) of the study period; We selected its natural cubic spline with df varying between 2 and 10, based on the quasi-Akaike information criterion (QAIC), and a value of 3 was finally chosen to control for long-term temporal trends (e.g., population aging and improvements in healthcare) (13); sine and cosine trigonometric terms were also included to adequately capture seasonal cycles; We used Dow as a categorical variable for weekday; and offset [log(pop_*t*_)] is an offset term using the logarithm of the annual registered population, which converts the model from estimating death counts to estimating mortality rates, thereby adjusting for the impact of population growth in Minhang District over the study period (11, 12, 14).

We constructed cross-basis (8) functions for each meteorological factor to fit its exposure-lag-response association with daily cancer mortality. We selected model parameters using the quasi-Akaike information criterion (QAIC). Specifically, we set the natural cubic splines with 4 df for the exposure dimension, 3 df for the lag dimension, and 7 df for time. Following previous work (15), we specified a maximum lag of 0–21 days to capture the delayed effects of extreme weather conditions on cancer mortality. The maximum lag period was selected based on both biological plausibility and statistical fit. We compared candidate models with maximum lags of 14, 21, and 30 days and lag dimension degrees of freedom of 2, 3, and 4 using the Quasi-Akaike Information Criterion (QAIC), a standard criterion for model selection in quasi-Poisson time-series models. The 21-day lag with 3 degrees of freedom for the lag dimension yielded the lowest QAIC value (Supplementary Table S1) and was therefore selected as the primary specification. In the model, natural cubic splines for RH and DP were each assigned 3 df (16, 17).

After model fitting, the meteorological value linked to the lowest cancer mortality risk was taken as the baseline, defined as the minimum mortality risk level. Daily and cumulative (0–21 days) relative risks (RRs) with 95% confidence intervals (CIs) for cancer mortality were then calculated across different levels of each meteorological factor to describe their acute effects.

To assess the potential confounding effect of air pollution, we performed sensitivity analyses by sequentially adjusting for different sets of air pollutants using Shanghai city-level daily concentrations (Table 1: model 1: unadjusted; model 2: adjusted for PM2.5; model 3: adjusted for PM2.5, NO_2_, and O3; model 4: adjusted for all available pollutants including PM2.5, PM10, SO_2_, CO, NO_2_, and O3). The pollutant concentrations were included as covariates using natural cubic splines with 3 degrees of freedom to control for non-linear effects. In addition, we conducted stratified analyses by sex (male vs. female) and age groups (< 60, 60–74, and ≥75 years) using the same model specification to identify potential effect modifiers.

**Table 1:** Model specification strategies for adjusting for potential confounding by air pollution.

Model 1	Unadjusted
Model 2	Adjusted PM2.5
Model 3	Adjusted PM2.5/NO_2_/O_3_
Model 4	Adjusted all pollutants, including PM2.5/NO_2_/O_3_/PM10/S0_2_/CO

## 3 Results

### 3.1 Characteristics of cancer deaths in Minhang district, 2014–2023

#### 3.1.1 Overall epidemiologic features

The study spanned 10 years (3,652 days). During this period, 26,684 cancer deaths were recorded in Minhang District: 62.26% (16,614/26,684) in males and 37.74% (10,070/26,684) in females. The daily mean was 7.31 ± 2.92 deaths. The mean age at death was 72.86 ± 12.45 years. Among all cancer decedents, the proportion of individuals aged < 60, 60–74, and ≥75 years was 13.11, 39.00, and 47.89%, respectively (Table 2).

**Table 2:** Baseline demographic characteristics of the study population (cancer decedents) in Minhang District, Shanghai, 2014–2023.

Characteristic	Value
**Total cancer deaths**, ***n***	26,684
**Daily mean deaths, mean** **±SD**	7.31 ± 2.92
Sex, *n* (%)	
Male	16,614 (62.26)
Female	10,070 (37.74)
**Age at death (years), mean** **±SD**	72.86 ± 12.45
Age group, *n* (%)	
< 60 years	3498 (13.11)
60–74 years	10406 (39.00)
≥75 years	12780 (47.89)
Major cancer types, *n* (%)	
Lung cancer	6,770 (25.37)
Colorectal cancer	3,302 (12.37)
Gastric cancer	2,958 (11.09)
Sex-specific ranking	
Male (1st → 3rd)	Lung > Gastric > Colorectal
Female (1st → 3rd)	Lung > Colorectal > Gastric

#### 3.1.2 Distribution characteristics of major cancer deaths

Between 2014 and 2023, lung cancer accounted for 25.37% of cancer deaths in Minhang District, followed by colorectal cancer (12.37%) and gastric cancer (11.09%). The rank order differed by sex: among males, lung cancer ranked first, followed by gastric cancer and then colorectal cancer; among females, the sequence was lung, colorectal, and gastric cancer (Table 2).

**Figure 1:**
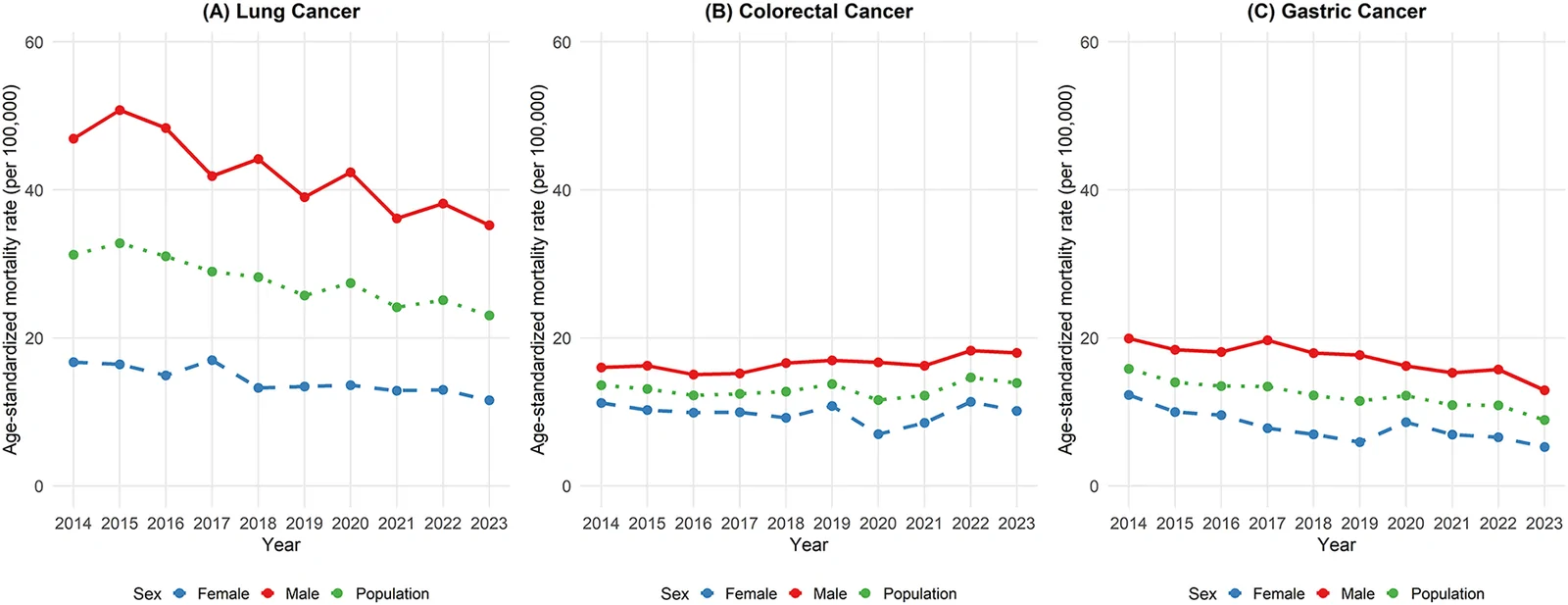
Trends in age-standardized mortality rates for major cancers in Minhang District, 2014–2023. **(A)** Lung cancer; **(B)** Colorectal cancer; **(C)** Gastric cancer. Blue lines represent females, red lines males, and green dotted lines population totals. APC, annual percent change. APCs from Joinpoint regression: lung cancer-males: −3.64% *, females: −3.78% *, total: −3.64% *; colorectal cancer-males: 1.57% *, females: −1.12% (*P* = 0.56), total: 0.51% (*P* = 0.66); gastric cancer-males: −3.83% *, females: −7.05% *, total: −4.93% *. *P*<*0.05* is indicated by an asterisk (*).

During the study period, the lung cancer age-standardized mortality rate showed a declining trend. The annual percent changes (APCs) stood at −3.64% for males, −3.78% for females, and −3.64% for the combined population (all *P* < 0.05). For colorectal cancer, the picture varied by sex: the rate rose in males (APC = 1.57%, *P* < 0.05), showed little change overall (APC = 0.51%, *P* = 0.66), and fell in females (APC = −1.12%, *P* = 0.56). Gastric cancer mortality declined across all groups, with APCs of −3.83% (males), −7.05% (females), and −4.93% (combined), all statistically significant (*P* < 0.05) (Figure 1).

### 3.2 Daily meteorological factors in Minhang District, 2014–2023

Across the study period, the daily mean temperature for Minhang District was 17.91 ± 8.57 °C, with values ranging from −5.40 °C to 35.20 °C. The daily minimum temperature averaged 14.50 ± 8.85 °C, falling between −7.40 and 30.50 °C. Mean daily relative humidity stood at 72.25 ± 13.40%, with a recorded range of 10.00–99.00% (Table 3).

**Table 3:** Distribution of meteorological factors in Minhang District, Shanghai, 2014–2023.

Meteorological variable	Mean ±*SD*	Minimum	*P*_25_	*P*_50_	*P*_75_	Maximum
Daily mean temperature (°C)	17.91 ± 8.57	−5.40	10.50	18.60	24.90	35.20
Daily maximum temperature (°C)	22.23 ± 8.82	−3.80	15.00	23.10	29.20	40.80
Daily minimum temperature (°C)	14.50 ± 8.85	−7.40	7.10	14.90	22.20	30.50
Diurnal temperature range (°C)	7.73 ± 3.45	0.70	5.10	7.50	10.10	20.40
Daily mean relative humidity (%)	72.25 ± 13.40	10.00	64.00	73.00	82.00	99.00
Daily precipitation (mm)	4.16 ± 11.77	0.00	0.00	0.00	2.10	200.60
Daily mean atmospheric pressure (hPa)	1015.93 ± 9.05	985.50	1008.30	1016.10	1023.10	1041.80
Daily mean wind speed (m/s)	1.56 ± 0.59	0.20	1.10	1.50	1.90	4.40

### 3.3 Correlation between daily major cancer deaths and meteorological factors

As a preliminary exploration, Spearman correlation analysis was used to assess the overall linear relationships, which showed no significant associations (Table 4). This finding is not unexpected, as the correlations between meteorological factors and mortality are often non-linear and lagged, which cannot be captured by simple correlation analysis.

**Table 4:** Spearman's rank correlation analysis between meteorological factors and daily cancer mortality in Minhang District, 2014–2023.

Cancer type	Lung cancer		Colorectal cancer		Gastric cancer	
meteorological factor	**r*_*s*_*	*P*	**r*_*s*_*	*P*	**r*_*s*_*	*P*
Daily mean temperature (°C)	−0.003	0.858	−0.019	0.262	−0.008	0.633
Daily maximum temperature (°C)	−0.002	0.910	−0.018	0.271	−0.005	0.754
Daily minimum temperature (°C)	−0.005	0.762	−0.020	0.220	−0.009	0.582
Diurnal temperature range (°C)	0.002	0.920	0.006	0.724	0.015	0.371
Daily mean relative humidity (%)	−0.007	0.691	0.009	0.589	−0.027	0.102
Daily precipitation (mm)	−0.001	0.959	0.011	0.512	−0.029	0.093
Daily mean atmospheric pressure (hPa)	0.012	0.470	0.020	0.235	0.023	0.160
Daily mean wind speed (m/s)	−0.011	0.519	−0.048	0.004	−0.003	0.864

### 3.4 Acute effects of meteorological factors on major cancer mortality

#### 3.4.1 Overall effects of different meteorological factors on daily major cancer mortality

**Figure 2:**
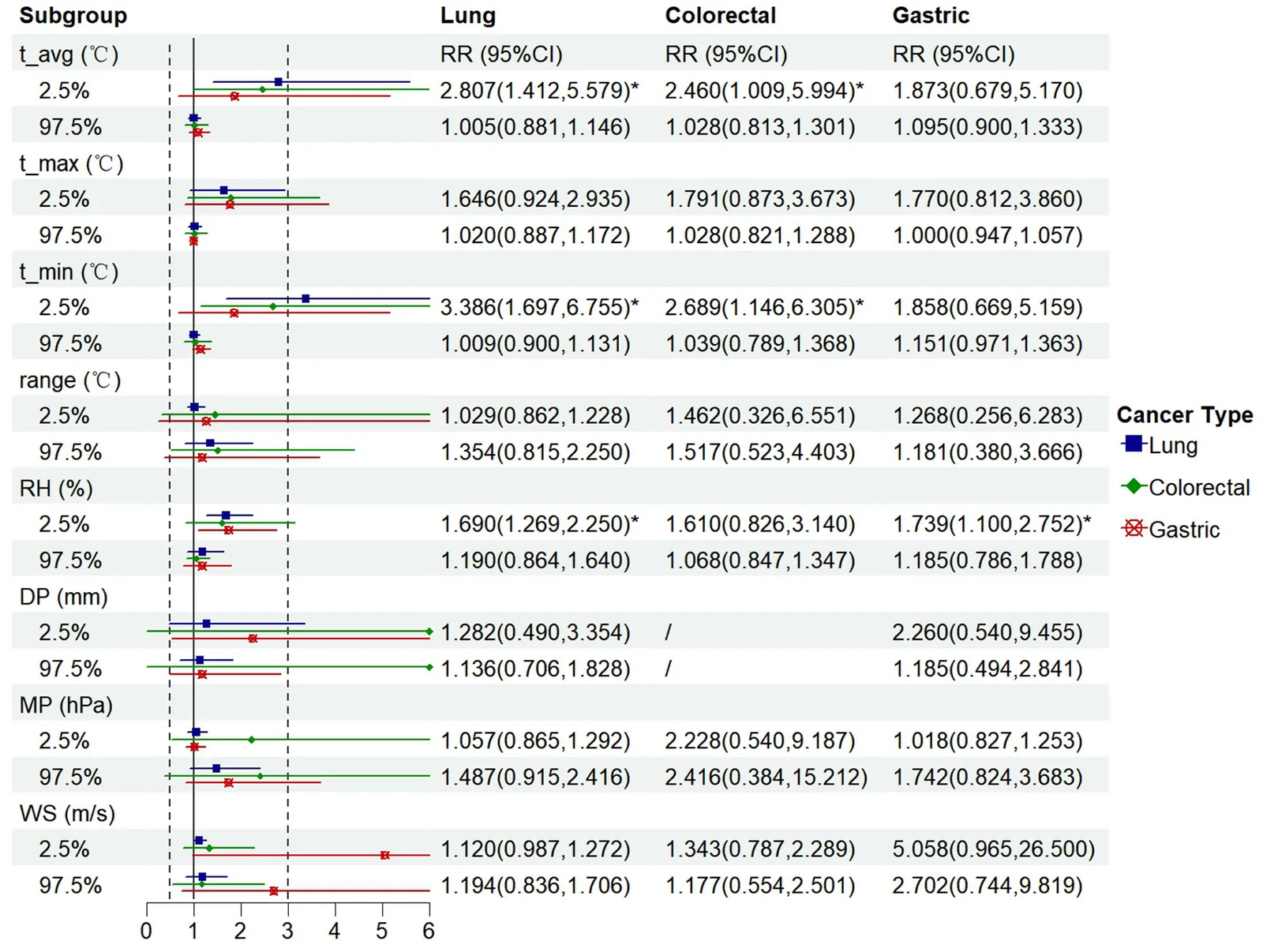
Forest plot of the association between extreme meteorological factors (2.5th and 97.5th percentiles) and cumulative mortality risk for major cancers. t_avg, daily mean temperature (°C); t_max, daily maximum temperature (°C); t_min, daily minimum temperature (°C); range, diurnal temperature range (°C); RH, relative humidity(%); DP, daily precipitation (mm); MP, mean atmospheric pressure (hPa); WS, wind speed(m/s). * indicates *P*<*0.05*.

Figure 2 summarizes the main findings. Using a 0–21 day cumulative lag window, we defined extreme weather as values falling below the 2.5th percentile or exceeding the 97.5th percentile, and linked these conditions to elevated daily mortality risk from major cancers. Among all factors examined, low temperature yielded the strongest statistical signal. At the lower extreme (2.5th percentile) of daily mean temperature, the RR for lung cancer stood at 2.807 (95% CI: 1.412–5.579) and at 2.460 (95% CI: 1.009–5.994) for colorectal cancer. At the same percentile of daily minimum temperature, the RR climbed to 3.386 (95% CI: 1.697–6.755) for lung cancer and to 2.689 (95% CI: 1.146–6.305) for colorectal cancer. At the 2.5th percentile of relative humidity, lung cancer had an RR of 1.690 (95% CI: 1.269–2.250), whereas gastric cancer had an RR of 1.739 (95% CI: 1.100–2.752). None of the remaining meteorological factors showed a statistically meaningful link to mortality from these cancers. On the whole, extreme weather conditions influenced lung cancer risk more strongly than colorectal or gastric cancer risk (Figure 2).

#### 3.4.2 Exposure–response relationship between major meteorological factors and daily lung cancer mortality

Among all cancer types examined, lung cancer showed the largest risk elevation under extreme weather conditions. Daily mean and minimum temperatures, along with relative humidity, were significantly associated with lung cancer mortality. Therefore, we focused the remaining analysis on lung cancer to evaluate the lag influences of these three meteorological variables.

##### 3.4.2.1 Mean temperature-mortality risk at different lag days

**Figure 3:**
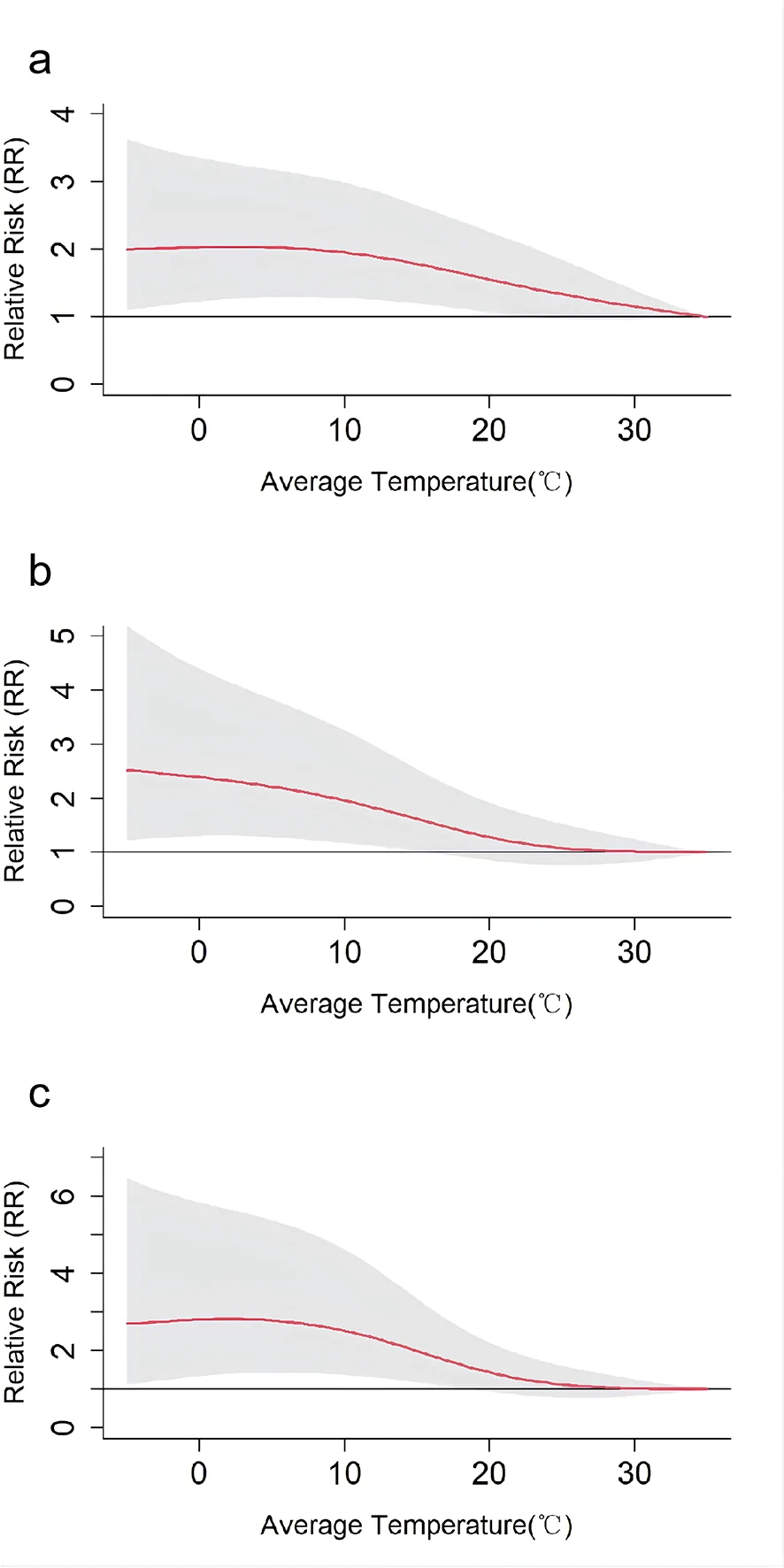
Daily mean temperature-lung cancer mortality risk at lag days of 7, 14, and 21 days. **(a**) Lag day, 7 days. **(b**) Lag day, 14 days. **(c**) Lag day, 21 days. The solid lines represent the estimated relative risks, and the shaded areas represent the 95% confidence intervals.

Across lag days, Figures 3a–c depict the variation in the association of mean daily temperature with lung cancer mortality risk. At lags of 7, 14, and 21 days, lower mean temperature consistently predicted higher lung cancer mortality. Moreover, the cumulative risk of lung cancer death further increased with longer lag times. At 3 °C (the 2.5th percentile of daily mean temperature), the RR for lung cancer mortality was estimated as 2.034 (95% CI: 1.275–3.245) for a 7-day lag, 2.297 (95% CI: 1.302–4.053) for 14 days, and 2.807 (95% CI: 1.412–5.579) for 21 days (Figure 3).

##### 3.4.2.2 Minimum temperature-mortality risk at different lag days

**Figure 4:**
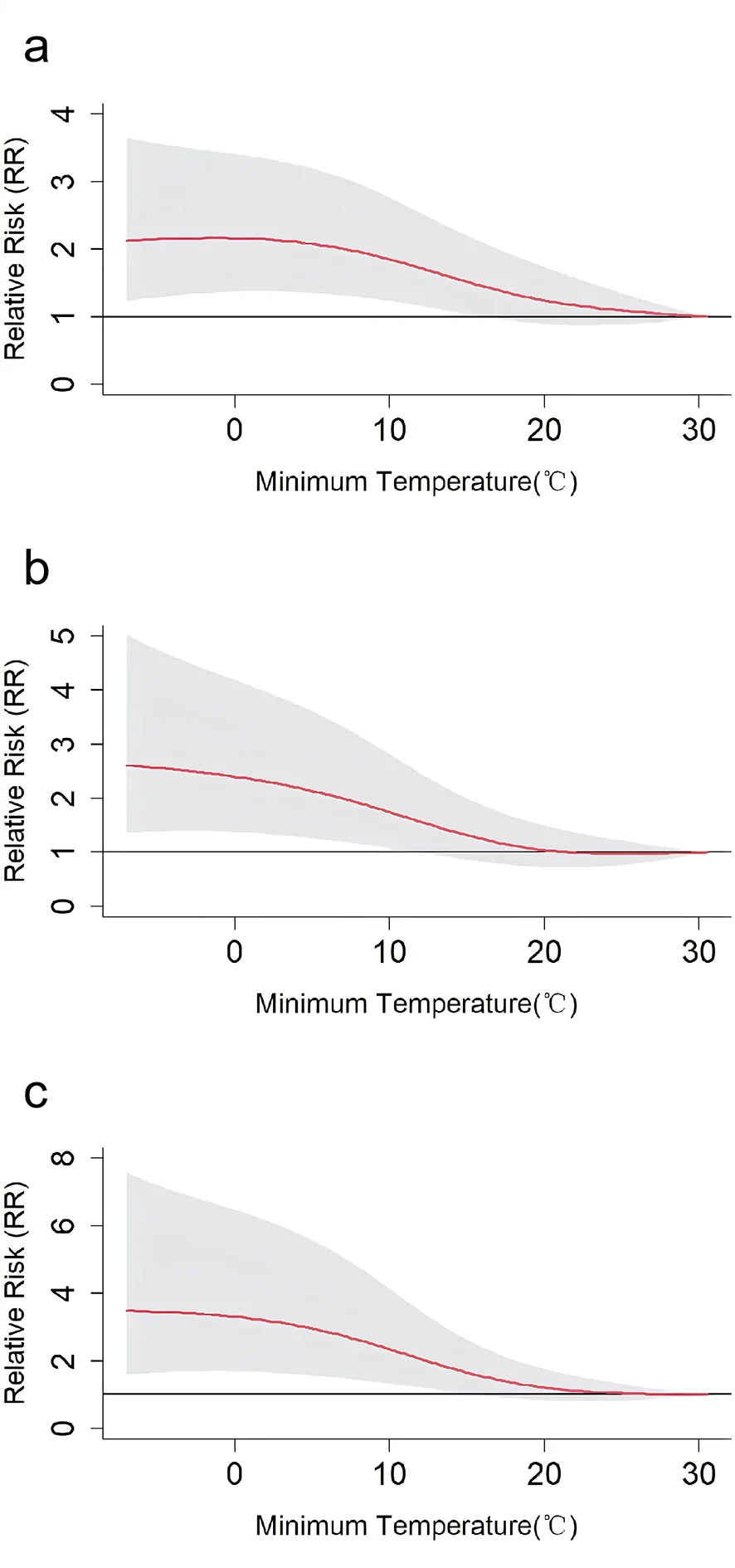
Daily minimum temperature-lung cancer mortality risk at lag days of 7, 14, and 21 days. **(a**) Lag, 7 days. **(b**) Lag, 14 days. **(c**) Lag, 21 days. The solid lines indicate the point estimates, and the shaded regions represent the 95% CIs.

Figures 4a–c show how the association of daily minimum temperature with lung cancer mortality risk varies across lag days. At lag days of 7, 14, and 21, a lower daily minimum temperature consistently corresponded to elevated lung cancer mortality risk. Furthermore, cumulative mortality risk increased progressively with longer lag times. At a daily minimum temperature of −2 °C (the 2.5th percentile), the RR of lung cancer mortality stood at 2.163 (95% CI: 1.349–3.470) with a 7-day lag, climbed to 2.475 (95% CI: 1.392–4.401) with a 14-day lag, and reached 3.386 (95% CI: 1.697–6.755) with a 21-day lag (Figure 4).

##### 3.4.2.3 Relative humidity-mortality risk at different lag days

**Figure 5:**
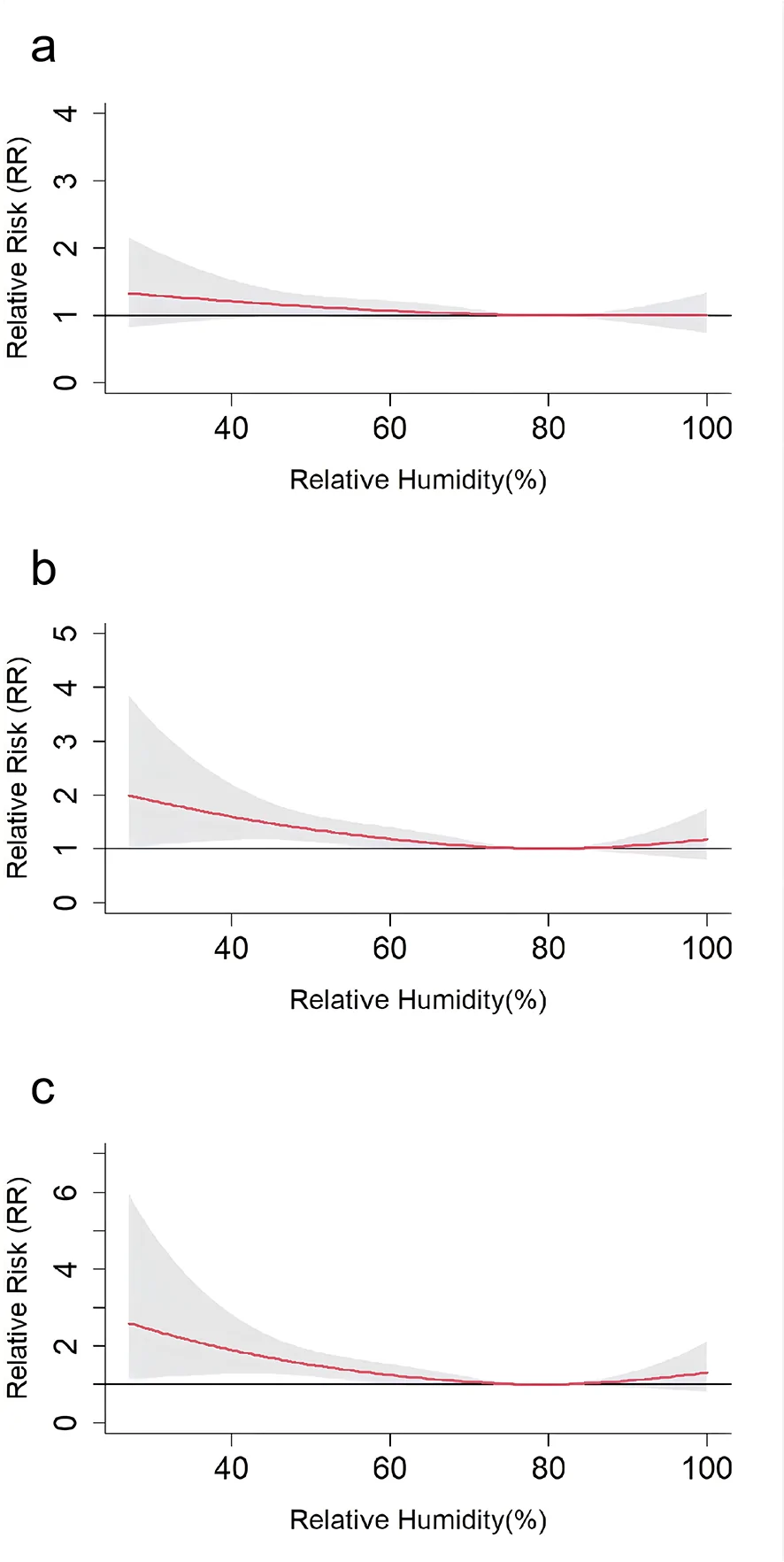
Daily relative humidity-lung cancer mortality risk at lag days of 7, 14, and 21 days. **(a**) Lag = 7 days. **(b**) Lag = 14 days. **(c**) Lag = 21 days. Solid lines indicate the estimated relative risks; shaded areas denote the 95% confidence intervals.

Figures 5a–c show the exposure-response associations and lag patterns linking daily relative humidity to lung cancer mortality. The effect of relative humidity on daily lung cancer death risk was mainly confined to the 30–60% range. At lag days of 7, 14, and 21, higher relative humidity consistently corresponded to a lower lung cancer mortality risk, indicating that the risk decreased with rising humidity. At a relative humidity of 45% (the 2.5th percentile), the RR of cancer mortality stood at 1.168 (95% CI: 0.982–1.388) with a 7-day lag, rose to 1.477 (95% CI: 1.172–1.861) with a 14-day lag, and reached 1.690 (95% CI: 1.269–2.250) with a 21-day lag (Figure 5).

### 3.5 Sensitivity analysis: adjustment for air pollution

To evaluate whether the observed associations were confounded by air pollution, we sequentially adjusted for different sets of air pollutants. As shown in Table 5, the cumulative RRs for lung cancer mortality associated with low temperature and low humidity remained statistically significant and were substantially unchanged after adjusting for PM2.5 alone (Model 2), PM2.5+NO_2_+O3 (Model 3), or all available pollutants (Model 4). For example, at the 2.5th percentile of daily mean temperature, the RR for lung cancer was 2.807 (95% CI: 1.412–5.579) in the unadjusted model, 2.788 (1.409–5.514) after adjusting for PM2.5, 2.832 (1.402–5.717) after adjusting for PM2.5+NO_2_+O3, and 2.949 (1.437–6.052) after adjusting for all pollutants. Similarly, for daily minimum temperature at the 2.5th percentile, the RRs were 3.386 (1.697–6.755), 3.303 (1.653–6.602), 3.397 (1.683–6.855), and 3.445 (1.669–7.108), respectively. These findings indicate that the associations between low temperature/low humidity and lung cancer mortality were robust to adjustment for air pollution.

**Table 5:** Sensitivity analysis of the association between extreme meteorological exposures and cumulative cancer mortality risk, comparing different air pollution adjustment strategies.

Variable	Model	Percentile	Lung	Colorectal	Gastric
Range	Model 1	2.50%	1.029 (0.862,1.228)	1.462 (0.326,6.551)	1.268 (0.256,6.283)
		97.50%	1.354 (0.815,2.25)	1.517 (0.523,4.403)	1.181 (0.38,3.666)
	Model 2	2.50%	1.033 (0.866,1.234)	1.462 (0.326,6.551)	1.268 (0.256,6.283)
		97.50%	1.354 (0.815,2.25)	1.517 (0.523,4.403)	1.181 (0.38,3.666)
	Model 3	2.50%	1.028 (0.859,1.231)	1.506 (0.335,6.772)	1.325 (0.267,6.581)
		97.50%	1.375 (0.824,2.295)	1.544 (0.53,4.498)	1.218 (0.391,3.791)
	Model 4	2.50%	1.026 (0.856,1.23)	1.506 (0.335,6.772)	1.325 (0.267,6.581)
		97.50%	1.313 (0.783,2.202)	1.544 (0.53,4.498)	1.218 (0.391,3.791)
t_avg	Model 1	2.50%	2.807 (1.412,5.579)^*^	2.46 (1.009,5.994)^*^	1.873 (0.679,5.17)
		97.50%	1.005 (0.881,1.146)	1.028 (0.813,1.301)	1.095 (0.9,1.333)
	Model 2	2.50%	2.788 (1.409,5.514)^*^	2.46 (1.009,5.994)^*^	1.873 (0.679,5.17)
		97.50%	1.003 (0.899,1.119)	1.028 (0.813,1.301)	1.095 (0.9,1.333)
	Model 3	2.50%	2.832 (1.402,5.717)^*^	2.388 (0.987,5.776)	1.882 (0.675,5.246)
		97.50%	1.007 (0.88,1.153)	1.063 (0.803,1.407)	1.098 (0.9,1.341)
	Model 4	2.50%	2.949 (1.437,6.052)^*^	2.388 (0.987,5.776)	1.882 (0.675,5.246)
		97.50%	1.009 (0.879,1.158)	1.063 (0.803,1.407)	1.098 (0.9,1.341)
t_max	Model 1	2.50%	1.646 (0.924,2.935)	1.791 (0.873,3.673)	1.77 (0.812,3.86)
		97.50%	1.02 (0.887,1.172)	1.028 (0.821,1.288)	1 (0.947,1.057)
	Model 2	2.50%	1.635 (0.916,2.917)	1.791 (0.873,3.673)	1.77 (0.812,3.86)
		97.50%	1.019 (0.887,1.172)	1.028 (0.821,1.288)	1 (0.947,1.057)
	Model 3	2.50%	1.646 (0.908,2.984)	1.788 (0.876,3.649)	1.729 (0.803,3.72)
		97.50%	1.021 (0.884,1.178)	1.05 (0.806,1.368)	1.006 (0.838,1.207)
	Model 4	2.50%	1.681 (0.922,3.067)	1.788 (0.876,3.649)	1.729 (0.803,3.72)
		97.50%	1.019 (0.879,1.18)	1.05 (0.806,1.368)	1.006 (0.838,1.207)
t_min	Model 1	2.50%	3.386 (1.697,6.755)^*^	2.689 (1.146,6.305)^*^	1.858 (0.669,5.159)
		97.50%	1.009 (0.9,1.131)	1.039 (0.789,1.368)	1.151 (0.971,1.363)
	Model 2	2.50%	3.303 (1.653,6.602)^*^	2.689 (1.146,6.305)^*^	1.858 (0.669,5.159)
		97.50%	1.007 (0.898,1.129)	1.039 (0.789,1.368)	1.151 (0.971,1.363)
	Model 3	2.50%	3.397 (1.683,6.855)^*^	2.554 (1.103,5.916)^*^	1.939 (0.693,5.422)
		97.50%	1.009 (0.899,1.133)	1.075 (0.781,1.479)	1.16 (0.978,1.376)
	Model 4	2.50%	3.445 (1.669,7.108)^*^	2.554 (1.103,5.916)^*^	1.939 (0.693,5.422)
		97.50%	1.015 (0.902,1.143)	1.075 (0.781,1.479)	1.16 (0.978,1.376)
RH	Model 1	2.50%	1.69 (1.269,2.25)^*^	1.61 (0.826,3.14)	1.739 (1.1,2.752)^*^
		97.50%	1.19 (0.864,1.64)	1.068 (0.847,1.347)	1.185 (0.786,1.788)
	Model 2	2.50%	1.67 (1.253,2.224)^*^	1.61 (0.826,3.14)	1.739 (1.1,2.752)^*^
		97.50%	1.189 (0.863,1.639)	1.068 (0.847,1.347)	1.185 (0.786,1.788)
	Model 3	2.50%	1.699 (1.269,2.274)^*^	1.555 (0.796,3.037)	1.715 (1.083,2.715)^*^
		97.50%	1.203 (0.87,1.664)	1.059 (0.839,1.335)	1.179 (0.779,1.785)
	Model 4	2.50%	1.655 (1.226,2.232)^*^	1.555 (0.796,3.037)	1.715 (1.083,2.715)^*^
		97.50%	1.245 (0.891,1.739)	1.059 (0.839,1.335)	1.179 (0.779,1.785)
DP	Model 1	2.50%	1.282 (0.49,3.354)	/	2.26 (0.54,9.455)
		97.50%	1.136 (0.706,1.828)	/	1.185 (0.494,2.841)
	Model 2	2.50%	1.35 (0.51,3.574)	/	2.26 (0.54,9.455)
		97.50%	1.123 (0.71,1.776)	/	1.185 (0.494,2.841)
	Model 3	2.50%	1.231 (0.457,3.313)	/	2.309 (0.548,9.74)
		97.50%	1.126 (0.683,1.856)	/	1.189 (0.493,2.866)
	Model 4	2.50%	1.38 (0.463,4.116)	/	2.309 (0.548,9.74)
		97.50%	1.039 (0.73,1.48)	/	1.189 (0.493,2.866)
MP	Model 1	2.50%	1.057 (0.865,1.292)	2.228 (0.54,9.187)	1.018 (0.827,1.253)
		97.50%	1.487 (0.915,2.416)	2.416 (0.384,15.212)	1.742 (0.824,3.683)
	Model 2	2.50%	1.055 (0.863,1.291)	2.228 (0.54,9.187)	1.018 (0.827,1.253)
		97.50%	1.48 (0.911,2.405)	2.416 (0.384,15.212)	1.742 (0.824,3.683)
	Model 3	2.50%	1.076 (0.866,1.337)	2.223 (0.534,9.249)	1.025 (0.813,1.294)
		97.50%	1.463 (0.903,2.372)	2.381 (0.375,15.119)	1.806 (0.859,3.798)
	Model 4	2.50%	1.077 (0.865,1.341)	2.223 (0.534,9.249)	1.025 (0.813,1.294)
		97.50%	1.463 (0.899,2.38)	2.381 (0.375,15.119)	1.806 (0.859,3.798)
WS	Model 1	2.50%	1.12 (0.987,1.272)	1.343 (0.787,2.289)	5.058 (0.965,26.5)
		97.50%	1.194 (0.836,1.706)	1.177 (0.554,2.501)	2.702 (0.744,9.819)
	Model 2	2.50%	1.154 (0.387,3.438)	1.343 (0.787,2.289)	5.058 (0.965,26.5)
		97.50%	1.218 (0.519,2.861)	1.177 (0.554,2.501)	2.702 (0.744,9.819)
	Model 3	2.50%	1.202 (0.402,3.589)	1.372 (0.804,2.344)	5.39 (0.924,28.359)
		97.50%	1.243 (0.529,2.925)	1.246 (0.584,2.658)	2.766 (0.759,10.076)
	Model 4	2.50%	1.275 (0.423,3.846)	1.372 (0.804,2.344)	5.39 (0.924,28.359)
		97.50%	1.319 (0.557,3.122)	1.246 (0.584,2.658)	2.766 (0.759,10.076)

t_avg, daily mean temperature (°C); t_max, daily maximum temperature (°C); t_min, daily minimum temperature (°C); range, diurnal temperature range (°C); RH, relative humidity(%); DP, daily precipitation (mm); MP, mean atmospheric pressure (hPa); WS, wind speed(m/s). ^*^ indicates *P*<*0.05*.

### 3.6 Stratified analyses by sex and age

To identify vulnerable subpopulations, we performed stratified analyses by sex and age group.

#### 3.6.1 Sex stratification

As shown in Table 6, the cold effect on lung cancer mortality was more pronounced in males than in females. At the 2.5th percentile of daily minimum temperature (t_min), the cumulative RR was 2.184 (95% CI: 1.208–3.947) in males, compared to 1.611 (95% CI: 0.958–2.708) in females. For relative humidity at the 2.5th percentile, the RR was 1.643 (95% CI: 1.285–2.101) in males, versus 1.072 (95% CI: 0.606–1.896) in females. These results suggest that males are more susceptible to cold- and low-humidity-related lung cancer mortality.

**Table 6:** Sex-stratified analysis: cumulative relative risks (RRs) of lung cancer mortality associated with extreme meteorological factors (2.5th and 97.5th percentiles).

Variable	Percentile	Male	Female
Range	2.50%	1.03 (0.74,1.434)	1.07 (0.911,1.256)
	97.50%	1.268 (0.918,1.752)	1.125 (0.712,1.777)
t_avg	2.50%	1.733 (0.959,3.13)	1.943 (1.109,3.405)^*^
	97.50%	1.007 (0.897,1.131)	1.106 (0.908,1.346)
t_max	2.50%	1.207 (0.732,1.991)	1.601 (1.011,2.535)^*^
	97.50%	1.022 (0.904,1.155)	1.072 (0.903,1.273)
t_min	2.50%	2.184 (1.208,3.947)^*^	1.611 (0.958,2.708)
	97.50%	1.016 (0.92,1.122)	1.143 (0.904,1.445)
RH	2.50%	1.643 (1.285,2.101)^*^	1.072 (0.606,1.896)
	97.50%	1.192 (0.91,1.56)	1.091 (0.466,2.552)
DP	2.50%	1.553 (0.661,3.648)	1.546 (0.663,3.606)
	97.50%	1.046 (0.74,1.477)	1.057 (0.725,1.541)
MP	2.50%	1.194 (0.94,1.517)	1.194 (0.939,1.518)
	97.50%	1.27 (0.863,1.868)	1.267 (0.861,1.864)
WS	2.50%	1.041 (0.935,1.16)	1.711 (0.63,4.647)
	97.50%	1.271 (0.937,1.724)	1.796 (0.827,3.904)

t_avg, daily mean temperature (°C); t_max, daily maximum temperature (°C); t_min, daily minimum temperature (°C); range, diurnal temperature range (°C); RH, relative humidity(%); DP, daily precipitation (mm); MP, mean atmospheric pressure (hPa); WS, wind speed(m/s). ^*^ indicates *P*<*0.05*.

#### 3.6.2 Age stratification

As presented in Table 7, the association between low temperature and lung cancer mortality was strongest in the 60–74 age group, with an RR of 2.104 (95% CI: 1.241–3.566) for daily mean temperature at the 2.5th percentile and 2.229 (95% CI: 1.349–3.683) for daily minimum temperature at the 2.5th percentile. The associations in the < 60 and ≥75 age groups were weaker and not statistically significant. For low humidity (RH at 2.5th percentile), both the 60–74 group (RR = 1.617, 95% CI: 1.264–2.068) and the ≥75 group (RR = 1.301, 95% CI: 1.004–1.687) showed elevated risks, while the < 60 group did not. These findings indicate that middle-aged and older adults (particularly those aged 60–74) are the most vulnerable to cold-related lung cancer mortality, whereas people aged 75 years or older also show susceptibility to low-humidity effects.

**Table 7:** Age-stratified analysis: cumulative relative risks (RRs) of lung cancer mortality associated with extreme meteorological factors (2.5th and 97.5th percentiles).

Variable	Percentile	< 60	60–75	≥75
range	2.50%	1.112 (0.947,1.307)	1.143 (0.782,1.67)	1.217 (0.887,1.669)
	97.50%	1.219 (0.764,1.943)	1.279 (0.903,1.813)	1.031 (0.72,1.476)
t_avg	2.50%	1.171 (0.778,1.763)	2.104 (1.241,3.566)^*^	1.278 (0.843,1.936)
	97.50%	1.179 (0.521,2.665)	1.06 (0.887,1.267)	1.035 (0.74,1.449)
t_max	2.50%	1.106 (0.707,1.729)	1.407 (0.911,2.175)	1.373 (0.846,2.228)
	97.50%	1.197 (0.558,2.569)	1.052 (0.881,1.256)	1 (0.983,1.017)
t_min	2.50%	1.158 (0.671,2)	2.229 (1.349,3.683)^*^	1.277 (0.925,1.762)
	97.50%	1.112 (0.867,1.426)	1.071 (0.886,1.293)	1.166 (0.72,1.889)
RH	2.50%	1.597 (0.886,2.88)	1.617 (1.264,2.068)^*^	1.301 (1.004,1.687)^*^
	97.50%	2.215 (0.913,5.373)	1.29 (0.983,1.692)	1.108 (0.831,1.477)
DP	2.50%	1.413 (0.611,3.265)	1.744 (0.678,4.488)	1 (1,1)
	97.50%	1.85 (0.303,11.3)	1.008 (0.805,1.262)	1.432 (0.571,3.59)
MP	2.50%	1.355 (0.889,2.064)	1.012 (0.747,1.371)	1.009 (0.829,1.227)
	97.50%	1.083 (0.793,1.478)	1.53 (0.88,2.169)	1.065 (0.681,1.664)
WS	2.50%	1.107 (0.97,1.264)	1.101 (0.415,2.918)	1.209 (0.856,1.709)
	97.50%	1.27 (0.919,1.756)	1.18 (0.553,2.518)	1.281 (0.789,2.08)

t_avg, daily mean temperature (°C); t_max, daily maximum temperature (°C); t_min, daily minimum temperature (°C); range, diurnal temperature range (°C); RH, relative humidity(%); DP, daily precipitation (mm); MP, mean atmospheric pressure (hPa); WS, wind speed(m/s). ^*^ indicates *P*<*0.05*.

### 3.7 Sensitivity analysis

To assess whether our findings were sensitive to the choice of lag structure and model parameters, we conducted sensitivity analyses by varying the maximum lag days (14, 21, and 30 days) and the degrees of freedom for the lag dimension (2, 3, and 4). As shown in Supplementary Table S2, the cumulative RRs for lung cancer mortality associated with low temperature (t_min at the 2.5th percentile) remained highly stable across all alternative specifications, with changes ranging from 0.24 to 4.49% relative to the main model. All 95% confidence intervals broadly overlapped with the main estimate, confirming the robustness of our findings.

## 4 Discussion

Between 2014 and 2023, the three leading causes of cancer death in Minhang District, Shanghai, were lung cancer, colorectal cancer, and gastric cancer. Males had a higher cancer mortality rate than females, which aligns with previous findings (18). Over the study period, the age-standardized mortality rates for lung and gastric cancers trended downward, while the change for colorectal cancer was not statistically significant.

We employed a DLNM to estimate the exposure–response associations of daily mean and minimum temperatures and relative humidity with major cancer mortality risk in Minhang District, Shanghai, across 2014–2023. The analysis revealed a clear nonlinear pattern linking these factors to the cumulative risk of daily lung cancer mortality. Importantly, the absence of significant linear correlation in the bivariate analysis does not contradict the DLNM results; rather, it highlights the added value of the DLNM approach in identifying non-linear exposure-lag-response relationships that would otherwise be masked in conventional correlation tests. Daily mean and minimum temperatures were also tied to cumulative colorectal cancer mortality. For gastric cancer, no meaningful associations emerged with any of the meteorological variables except for daily mean relative humidity. These results align with those of Zhang et al. (19) who reported that temperature changes affect cancer outcomes. The main estimates remained robust after we varied the maximum lag days and the df for the lag dimension. This observation indicates that our conclusions do not rely excessively on specific parameter choices, reflecting good robustness and credibility of the analysis.

In our analysis, we fitted a DLNM with a cross-basis function for daily mean temperature. After estimating the overall cumulative exposure-response association (0–21 day lag), we identified the minimum mortality temperature (MMT) as the temperature corresponding to the minimum of the fitted cumulative risk curve, based on the best linear unbiased predictions from the model. The lowest risk of lung cancer death occurred at a daily mean temperature of 29 °C. This value is slightly higher than the optimum temperature reported in other studies. The difference likely reflects variations in geographic setting, population makeup, and climate conditions (20). At daily mean temperatures below roughly 10 °C, lung cancer mortality risk increased sharply. Relative to the temperature associated with the minimum risk, a decline in mean temperature resulted in a gradual increase in daily lung cancer mortality, while a rise in mean temperature produced no comparable effect. These results imply that cold temperatures could carry a greater threat for lung cancer death, a conclusion that partly agrees with Chen et al. (21). Our results, however, differ from those of Zhang et al., who studied Chengdu. This inconsistency may stem from geographic contrasts between inland and coastal cities (22, 23). The results further revealed a notable lag effect of cold exposure. Across different levels of daily minimum temperature, a noticeable rise in lung cancer mortality risk was observed around lag day 3, with the risk continuing to increase as the lag period extended. These observations agree with those reported by Xiao et al. (24) in Hefei. When the daily minimum temperature rose above 15 °C (the 75th percentile), daily lung cancer death risk showed no meaningful change. Although the exact mechanisms through which ambient temperature influences lung cancer mortality remain unclear, it is generally accepted that low temperature can raise vascular resistance and blood pressure, thereby precipitating acute fatal events (25). This study further assessed how relative humidity affects both the cumulative and delayed risks of lung cancer death. The lowest cumulative relative risk was observed at 82% relative humidity, a result comparable to that reported in Wuhu City (6). Within the 30–60% relative humidity range, the daily risk of lung cancer death fell steadily as humidity levels rose.

The precise biological pathways through which weather conditions affect lung cancer death remain largely unclear. Although epidemiological studies on respiratory infections have demonstrated that cold and dry air can have detrimental effects on respiratory mucosal function, this mechanism alone is insufficient to explain the acute mortality effect observed in cancer patients (26). A more plausible interpretation is that meteorological exposure precipitates acute intercurrent events—such as pneumonia or respiratory failure (27)—in patients already at an advanced stage of cancer. Terminally ill cancer patients, particularly those with lung cancer, often have compromised pulmonary function, reduced immune competence (28), and limited physiological reserve. Exposure to cold and dry air may further impair mucociliary clearance and increase susceptibility to respiratory infections (29), triggering acute deteriorations in these vulnerable individuals. Importantly, when such events occur, death certificates are typically coded to the underlying malignancy (30, 31) rather than the immediate cause, which may introduce outcome misclassification. This interpretation is consistent with the harvesting hypothesis, suggesting that weather-related mortality displacement may primarily affect terminally ill patients by advancing death timing. This pathway fundamentally changes the interpretation of our findings: rather than suggesting that cold directly causes cancer death, our results are more consistent with a harvesting effect, where cold exposure advances the timing of death in patients already approaching the end of life due to their malignancy. This distinction is important for both biological plausibility and public health messaging.

The weaker associations observed for colorectal and gastric cancers, compared with lung cancer, may be explained by their longer disease trajectory, in which acute meteorological triggers are less likely to override the dominant influence of lifestyle and genetic factors. Our findings suggest that it may be important to distinguish between short-term weather fluctuations and long-term lifestyle and environmental exposures when examining cancer etiology. Climate change can also worsen air quality, particularly by raising PM2.5 levels, which has been widely recognized as a major risk factor for lung cancer development (32). A substantial body of evidence has identified long-term exposure to air pollution as a well-established risk factor for both lung and colorectal cancers (33–36). This long-term carcinogenic pathway differs from the acute triggering mechanism suggested by our short-term observations. Chronic carcinogenesis and the acute precipitation of terminal events may unfold on different time scales, and the present study primarily captures the latter through the short-term lagged associations observed. Future studies with longer lag windows or focusing on acute complications (e.g., obstruction, perforation) may be needed to detect weather-related effects.

In summary, this study used a DLNM to examine how various meteorological factors relate to mortality risk from major cancers. After accounting for temporal trends, air pollution, and weekday effects, low temperature and low humidity remained clearly linked to elevated lung cancer mortality within Minhang District. This lag effect lasted roughly 3 weeks. Our stratified analyses provide empirical support for prioritizing high-risk groups in early warning systems. Specifically, males and individuals aged 60–74 years exhibited the strongest associations between cold exposure and lung cancer mortality. These findings are consistent with previous studies reporting that males have higher occupational and lifestyle-related exposure burdens (37, 38), and that the 60–74 age group may represent a window of elevated vulnerability before competing risks from advanced age and comorbidities become dominant. Therefore, targeted public health interventions—such as community-based cold-wave alerts and home heating support—should prioritize these subgroups. Our findings support stronger collaboration between public health and meteorological authorities to enable timely community-based warnings and protective actions before the onset of extreme cold and low humidity. Vulnerable groups, particularly lung cancer patients, should be prioritized in these efforts. Additionally, integrating meteorological risk information into primary care practices could help health systems shift from reactive treatment toward proactive prevention, potentially reducing acute health events and their associated burden on the health system.

Several limitations of this study should be acknowledged. First, exposure to meteorological factors was assessed using data from a single monitoring station, which was then applied uniformly to all deaths in Minhang District. While this approach is common in time-series studies at the city or district level, it may introduce non-differential exposure misclassification if there is spatial heterogeneity in meteorological conditions across the district. However, Minhang District covers a relatively small geographic area (approximately 372 km^2^) and is an urban district with relatively homogeneous climatic conditions. Moreover, the monitoring station is a quality-controlled national meteorological station located centrally within the district. Nevertheless, this limitation would likely bias our effect estimates toward the null, meaning that our findings may represent conservative estimates of the true associations. We acknowledge that our air pollution adjustment used city-level rather than district-level monitoring data, which may have introduced non-differential exposure misclassification and potentially attenuated the effect estimates. Despite this limitation, the sensitivity analyses adjusting for various pollutant combinations yielded consistent results, supporting the robustness of our main findings. Future studies with fine-grained, district-specific air pollution data are warranted to further validate these results. Besides, we acknowledge that the ‘harvesting effect' (mortality displacement)—whereby an acute exposure may only advance the timing of death among frail individuals by a few days—could partially contribute to the observed associations. We also acknowledge that the sample size of this single-district study (26,684 deaths over 10 years, with a daily average of 7.31 deaths) limits the statistical power for stratified analyses. When stratifying by cancer type, meteorological factor, and lag days, the number of events in certain subgroups became small, resulting in wide confidence intervals. Although the DLNM framework helps to borrow information across lag days, the precision of some estimates should be interpreted with caution. Future studies with larger sample sizes or multi-center designs are warranted to confirm our findings. Finally, because of the ecological study design, we could not account for individual differences in exposure. We also lacked data on personal smoking or medication use, which may have introduced residual confounding.

Several directions warrant further investigation. First, while our sensitivity analyses using city-level air pollution data yielded consistent results, these findings should be validated using district-specific, high-resolution air pollution monitoring data to reduce exposure misclassification and obtain more precise effect estimates. Second, future studies with extended lag periods (e.g., up to 40 days) and larger sample sizes are warranted to explicitly quantify the extent of mortality displacement and to distinguish short-term harvesting from long-term causal effects. Third, future research should incorporate individual-level exposure assessment (e.g., personal monitoring, time-activity patterns) and detailed information on lifestyle factors (e.g., smoking, indoor heating, medication use) to better account for residual confounding and effect modification. Finally, given the heterogeneous effects observed across age and sex groups, larger multi-center studies with sufficient statistical power are needed to confirm the differential vulnerability patterns and to develop subgroup-specific early warning thresholds. Such efforts will not only strengthen causal inference but also facilitate the translation of our findings into precision public health interventions.

## 5 Conclusions

This study is the first to systematically assess the acute effects of meteorological factors on major cancer mortality in Minhang District, Shanghai. Our results show that exposure to low temperature and low humidity was linked to a significantly elevated lung cancer mortality risk, with the lagged effect persisting for up to 3 weeks. Our results highlight the need for greater attention to extreme weather conditions and the integration of weather information into disease prevention efforts. Weather-based health warnings targeting high-risk populations could help improve disease management.

## Data Availability

The raw data supporting the conclusions of this article will be made available by the authors, without undue reservation.
