# Supplementary material for: Association of meteorological exposures with major cancer mortality in Minhang District, Shanghai: a time series study based on distributed lag non-linear models
Source: Front Public Health. 2026 Aug 27;14:1923874. doi: 10.3389/fpubh.2026.1923874 (PMC13558136; doi:10.3389/fpubh.2026.1923874)
Supplement: Supplementary file 1 [file Data_Sheet_1.pdf]

List of Supplementary Tables

Supplementary Table S1. QAIC values for different lag structures (maximum lag days × degrees of freedom for lag dimension).

Supplementary Table S2. Sensitivity analysis of the cold effect on lung cancer mortality under different model specifications (varying maximum lag days and lag degrees of freedom).

Supplementary Table S1. QAIC values for different lag structures

Maximum lag days	df for lag dimension (2)	df for lag dimension (3)	df for lag dimension (4)
14	4139.426	4138.553	4142.611
21	4125.981	4125.000	4127.593
30	4131.616	4131.045	4134.632

Note: QAIC values calculated using daily mean temperature as the representative meteorological variable. Lower QAIC indicates better model fit.

Supplementary Table S2. Sensitivity analysis of the cold effect on lung cancer mortality under different model specifications

Scenario	Max lag (days)	df (lag)	RR (95% CI) for t_min at 2.5th percentile	Change vs. main model
Main model	21	3	3.386 (1.697, 6.755)	Reference
L=14	14	3	3.324 (1.712, 6.455)	1.83%
L=30	30	3	3.413 (1.744, 6.678)	0.80%
df_lag=2	21	2	3.538 (1.780, 7.033)	4.49%
df_lag=4	21	4	3.394 (1.740, 6.619)	0.24%

Note: All RRs are cumulative estimates over the specified lag period. None of the alternative specifications produced estimates that differed meaningfully from the main model (all 95% CIs overlapped broadly), confirming the robustness of our findings.
